# Supplementary material for: Cryo-EM structures of tau filaments from the brains of mice transgenic for human mutant P301S Tau
Source: Acta Neuropathol Commun. 2023 Oct 5;11:160. doi: 10.1186/s40478-023-01658-y (PMC10552433; doi:10.1186/s40478-023-01658-y)
Supplement: Supplementary file 1 — Supplementary Material 1 [file 40478_2023_1658_MOESM1_ESM.docx]

**Cryo-EM structures of Tau filaments from the**

**brains of mice transgenic for human mutant**

**P301S Tau**

Manuel Schweighauser^1^, Alexey G. Murzin^1^, Jennifer Macdonald^1^,

Isabelle Lavenir^1^, R. Anthony Crowther^1^, Sjors H.W. Scheres^1^* and

Michel Goedert^1^*

^1^Medical Research Council Laboratory of Molecular Biology, Cambridge, UK

*These authors jointly supervised the work. Correspondence to:

Sjors H.W. Scheres [scheres@mrc-lmb.cam.ac.uk](mailto:scheres@mrc-lmb.cam.ac.uk)

Michel Goedert [mg@mrc-lmb.cam.ac.uk](mailto:mg@mrc-lmb.cam.ac.uk)

**SUPPLEMENTARY TABLE**

**Table S1. Cryo-EM data acquisition and structure determination**

|  | Tg2541  24 weeks  EMD-18269  PDB 8Q96 | Tg2541  8 weeks | PS19  48 weeks  EMD-18268  PDB 8Q92 |
| --- | --- | --- | --- |
| **Data collection and processing** | |  |  |
| Magnification | 105,000 | 81,000 | 96,000 |
| Voltage (kV) | 300 | 300 | 300 |
| Detector | K2 Summit | K3 | Falcon 4 |
| Electron dose (e–/Å^2^) | 34.2 | 29.4 | 30.0 |
| Defocus range (μm) | 1.8-2.4 | 1.6-2.8 | 1.5-2.4 |
| Pixel size (Å)  Micrographs (no.) | 1.15  3,850 | 0.93  11,087 | 0.824  9,622 |
| Initial particle images (no.) | 192,394 | 167,465 | 265,469 |
| Symmetry imposed | C1 | C1 | C1 |
| Final particle images (no.) | 22,760 | 165,813 | 36,951 |
| Map resolution (Å)  FSC threshold = 0.143 | 3.09 | 4.29 | 3.05 |
| Helical rise (Å)  Helical twist (°) | 4.75  -0.83 | 4.75  -0.80 | 4.71  -0.98 |
|  |  |  |  |
|  | Tg2541  24 weeks |  | PS19  48 weeks |
| **Model Refinement** |  |  |  |
| Initial model used (PDB) | - |  | - |
| Model resolution (Å)  FSC threshold = 0.5 | 3.00 |  | 3.05 |
| Map sharpening *B* factor (Å^2^) | -55.3 |  | -48.1 |
| Model composition  Non-hydrogen atoms  Protein residues  Ligands | 2,100  312  0 |  | 2,091  282  0 |
| *B* factors (Å^2^)  Protein | 41.6 |  | 47.4 |
| R.m.s. deviations  Bond lengths (Å)  Bond angles (°) | 0.0064  1.143 |  | 0.0060  1.196 |
| Validation  MolProbity score  Clashscore  Poor rotamers (%) | 1.27  1.5  0.00 |  | 1.36  0.94  1.23 |
| Ramachandran plot  Favored (%)  Allowed (%)  Disallowed (%) | 94.44  5.56  0.00 |  | 91.30  8.7  0.00 |

**SUPPLEMENTARY FIGURES**

**
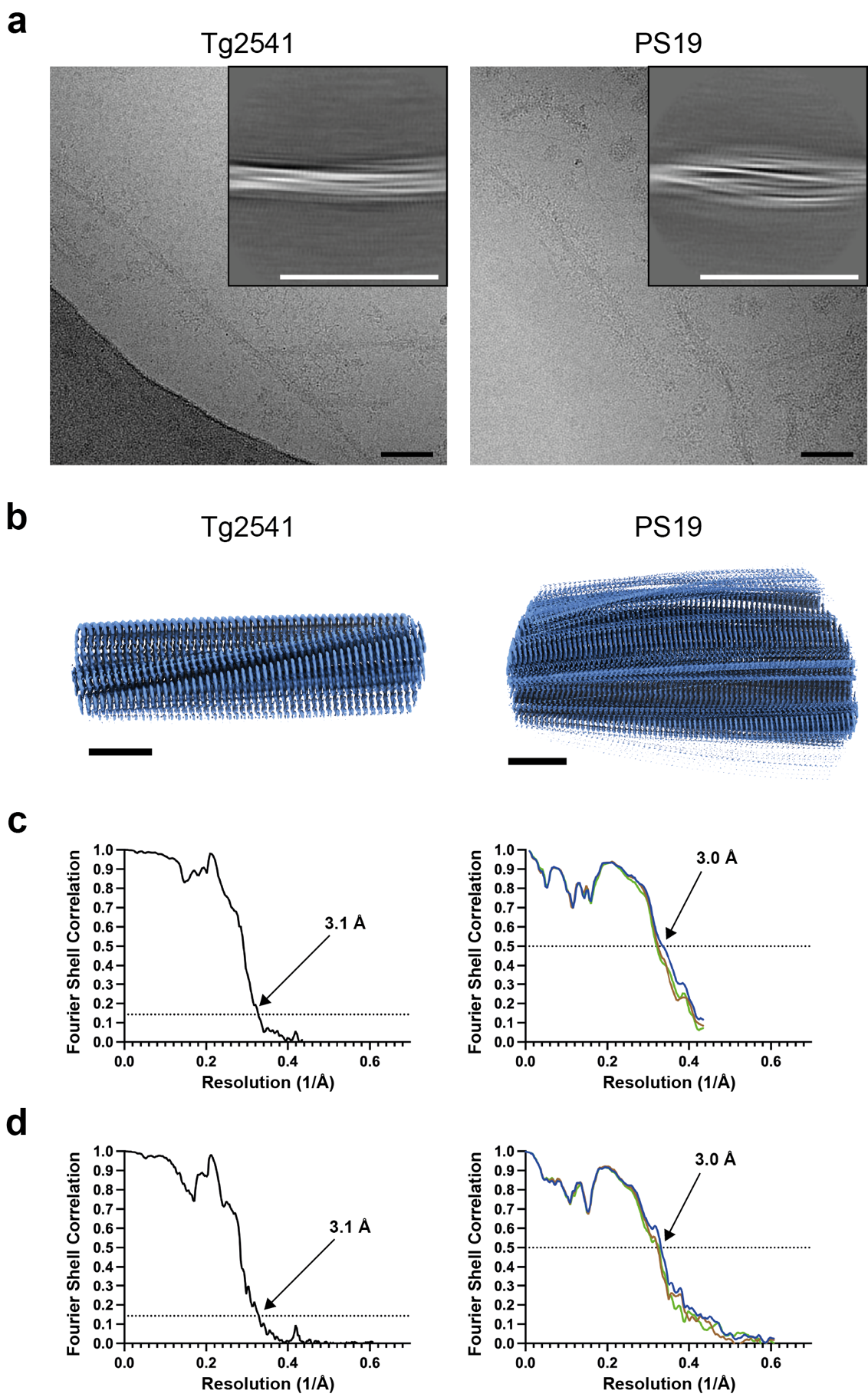
**

**Figure S1. Cryo-EM 2D classifications and resolution estimates**

**a,** Representative electron cryo-micrographs and 2D classification images (insets) of tau filaments from mouse lines Tg2541 and PS19 that are transgenic for human P301S tau.

b, 3D reconstructions of tau filaments from mouse lines Tg2541 and PS19. Scale bars, 5 nm.

**c,d,** Solvent-corrected Fourier shell correlation (FSC) curves of cryo-EM half-maps (left panels) and model-to-map validation (right panels) for P301S filaments from Tg2541 (b) and PS19 mice (c). FSC curves between the model refined in the combined map versus the combined map are shown in blue; FSC curves between a model refined in half-map 1 versus half-map 1 are shown in brown (model 1 versus half-map 1); FSC curves between the same model versus half-map 2 are shown in green (model 1 versus half-map 2).

**
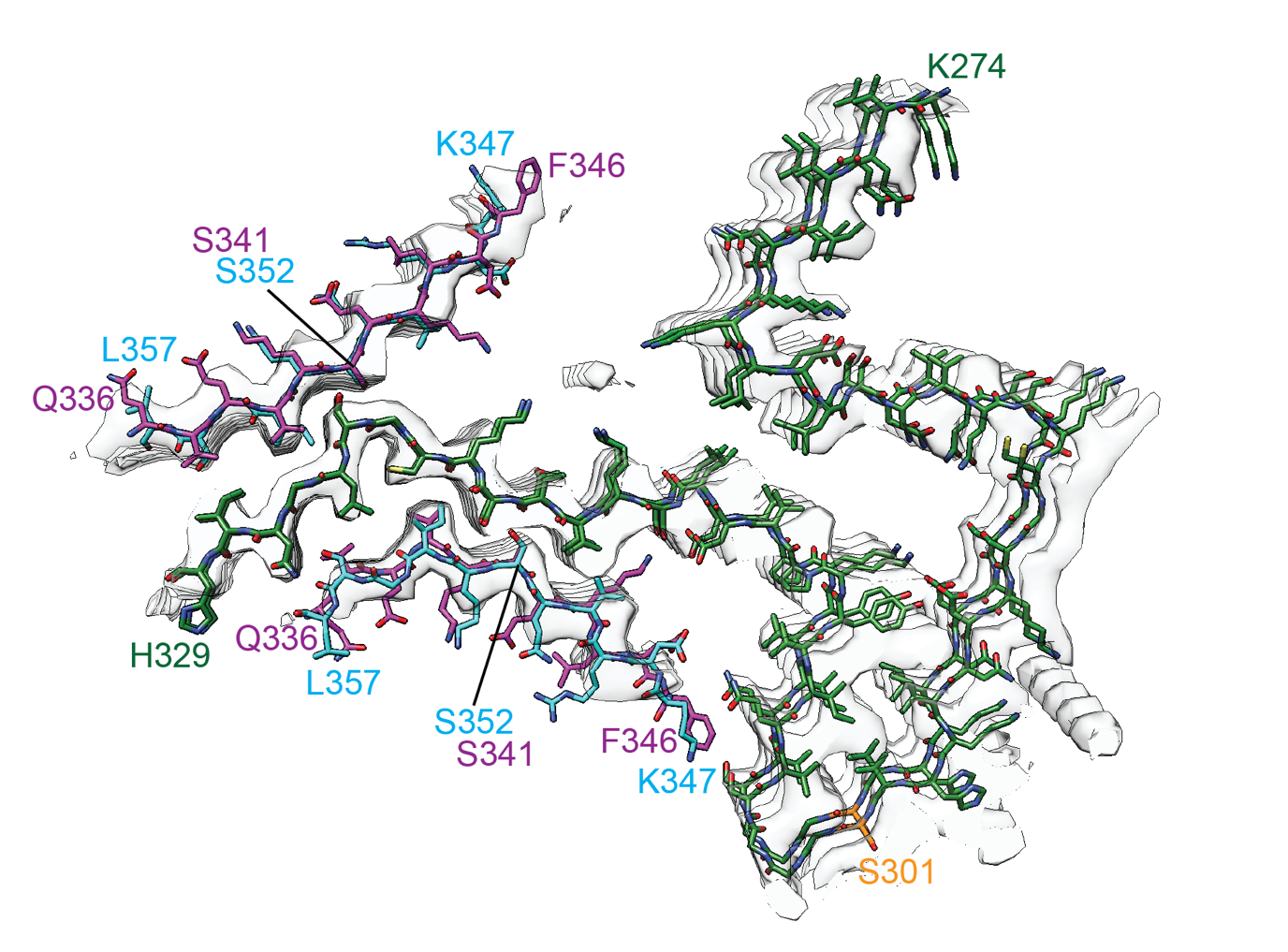
**

**Figure S2. Proposed model for islands A and B of the Tg2541 Tau filament fold**

The densities of both islands on two adjacent rungs were fitted with b-hairpins comprising tau residues 336-357. N-terminal strand 336-346 is shown in magenta and C-terminal strand 347-357 in cyan. Contiguous segment 274-329 is shown in green, with the mutation site P301S shown in orange. Residues S341 and S352 that are in ‘anchor’ positions are labelled.
